# Genetic heterogeneity in cholangiocarcinoma: a major challenge for targeted therapies

**DOI:** 10.18632/oncotarget.4539

**Published:** 2015-06-19

**Authors:** Giovanni Brandi, Andrea Farioli, Annalisa Astolfi, Guido Biasco, Simona Tavolari

**Affiliations:** ^1^ Department of Experimental, Diagnostic and Specialty Medicine, S. Orsola-Malpighi University Hospital, Bologna, Italy; ^2^ “G. Prodi” Interdepartmental Center for Cancer Research (C.I.R.C.), University of Bologna, Bologna, Italy; ^3^ Department of Medical and Surgical Sciences, S. Orsola-Malpighi University Hospital, Bologna, Italy; ^4^ Center for Applied Biomedical Research (C.R.B.A.), S. Orsola- Malpighi University Hospital, Bologna, Italy; ^5^ GICO- Italian Group of Cholangiocarcinoma, Italy

**Keywords:** cholangiocarcinoma, genetic heterogenity, targeted therapies

## Abstract

Cholangiocarcinoma (CC) encompasses a group of related but distinct malignancies whose lack of a stereotyped genetic signature makes challenging the identification of genomic landscape and the development of effective targeted therapies.

Accumulated evidences strongly suggest that the remarkable genetic heterogeneity of CC may be the result of a complex interplay among different causative factors, some shared by most human cancers while others typical of this malignancy.

Currently, considerable efforts are ongoing worldwide for the genetic characterization of CC, also using advanced technologies such as next-generation sequencing (NGS). Undoubtedly this technology could offer an unique opportunity to broaden our understanding on CC molecular pathogenesis. Despite this great potential, however, the high complexity in terms of factors potentially contributing to genetic variability in CC calls for a more cautionary application of NGS to this malignancy, in order to avoid possible biases and criticisms in the identification of candidate actionable targets. This approach is further justified by the urgent need to develop effective targeted therapies in this disease.

A multidisciplinary approach integrating genomic, functional and clinical studies is therefore mandatory to translate the results obtained by NGS into effective targeted therapies for this orphan disease.

## TO THE EDITORS

Cholangiocarcinoma (CC) is a complex disease encompassing a group of related but distinct malignancies that may emerge at different anatomic sites along the intrahepatic and extrahepatic biliary tree [1].

CC treatment represents a major challenge for oncologists. To date radical surgery, suitable only in a limited number of patients, has offered the only chance of cure [2]. Systemic treatment is confined to chemotherapy, whose front-line backbone is gemcitabine in combination with a platinum compound, while the efficacy of second-line remains to be established [3, 4].

The addition of targeted therapies in CC management was expected to broaden the therapy options and thereby improve the survival rate of these patients. However, targeted therapies have failed or shown only marginal benefits in several clinical trials with different drugs alone or combination with chemotherapy (Table 1 and 2) [5-18, reviewed in 19]. The development of effective targeted therapies in CC is challenging not only because most of the clinical trials have been designed for a mixed cohort of biliary tract cancer patients (thus frequently under-powering the impact of CC patients), but also because of the underlying genetic variability of the disease, whose lack of a stereotyped genetic signature makes difficult the identification of potential actionable targets [20].

**Table 1:** Clinical trails with tareted therapies, alone or in combination with chemotherapy, in CC

THERAPEUTIC REGIMEN	TARGET	PHASE	N° OF PATIENTS	END-POINTS: results (months)
SORAFENIB ^(5)^	VEGFR-2/-3, PDGFR-β, B-Raf, C-Raf	II	46	PFS: 2.3 OS: 4.4
SUNITINIB ^(6)^	VEGFR, PDGFR and KIT	II	56	TTP: 1.7
LAPATINIB ^(7)^	EGFR, Her2/Neu	II	57	PFS: 1.8 OS: 5.2
SELUMETINIB ^(8)^	MEK1, MEK2	II	88	PFS: 3.7 OS: 9.8
GEMOX + BEVACIZUMAB ^(9)^	VEGF	II	35	PFS : 7.0
GEMOX + CETUXIMAB ^(10)^	EGFR	II	76	PFS: 6.1 OS: 11.0
GEMOX + PANITUMUMAB ^(11)^	EGFR	II	46	PFS: 8.3 OS: 10.0
GEMOX + ERLOTINIB ^(12)^	EGFR	III	135	PFS: 5.8 OS: 9.5
DOCETAXEL + ERLOTINIB ^(13)^	EGFR	II	11	OS: 5.7
GEMCITABINE + CETUXIMAB ^(14)^	EGFR	II	44	OS: 13.5
BEVACIZUMAB + ERLOTINIB ^(15)^	VEGF, EGFR	II	49	OS: 9.9 TTP: 4.4
SORAFENIB + ERLOTINIB ^(16)^	VEGFR-2/-3, PDGFR-β, B-Raf, C-Raf, EGFR	II	34	PFS: 2.0 OS: 6.0
CISPLATIN/GEMCITABINE + SORAFENIB ^(17)^	VEGFR-2/-3, PDGFR-β, B-Raf, C-Raf	II	39	PFS: 6.5 OS: 14.4

Abbreviations: GEMOX, gemcitabine and oxaliplatin; PFS, progression-free survival; OS, overall survival; TTP, time to progression.

**Table 2:** Clinical trails with targeted therapies, alone or in combination with chemotherapy, currently in development in CC

THERAPEUTIC REGIMEN	TARGET	PHASE	ClinicalTrials.gov Identifier
TRASTUZUMAB	HER2/neu	II	NCT00478140
MK2206	AKT	II	NCT01425879
EVEROLIMUS	mTOR	II	NCT00973713
REGORAFENIB	VEGFR1-3, c-KIT, TIE-2, PDGFR-β, C-Raf, B-Raf, p38 MAPK, FGFR-1,	II	NCT02115542
GEMCITABINE + SORAFENIB	VEGFR-2/-3, PDGFR-β, B-Raf, C-Raf	II	NCT00661830
GEMOX + SORAFENIB	VEGFR-2/-3, PDGFR-β, B-Raf, C-Raf	I/II	NCT00955721
GEMOX + BINIMETINIB	MEK1, MEK2	I	NCT02105350
PANITUMUMAB + GEMCITABINE/IRINOTECAN	EGFR	II	NCT00948935
AFATINIB + GEMCITABINE/CISPLATIN	HER2/EGFR	I	NCT01679405
BINIMETINIB + GEMCITABINE/CISPLATIN	MEK1, MEK2	I/II	NCT01828034
EVEROLIMUS + GEMCITABINE/CISPLATIN	mTOR	I	NCT00949949
SELUMETINIB + GEMCITABINE/CISPLATIN	MEK1, MEK2	I/II	NCT01242605
CEDIRANIB + GEMCITABINE/CISPLATIN	VEGFR-1, VEGFR-2, VEGFR-3	II/III	NCT00939848
CEDIRANIB MALEATE + FOLFOX 6	VEGFR-1, VEGFR-2, VEGFR-3	II	NCT01229111
(GEMCITABINE, OXALIPLATIN, CAPECITABINE) + PANITUMUMAB OR BEVACIZUMAB	EGFR, VEGF	II	NCT01206049

Abbreviations: GEMOX, gemcitabine and oxaliplatin; nFOLFOX6, folinic acid-fluorouracil-oxaliplatin-6

Currently, considerable efforts are ongoing worldwide for the genetic characterization of CC, also using advanced technologies such as next-generation sequencing (NGS). NGS has opened the possibility to identify the full repertoire of tumor-borne genetic alterations [21, 22] and, to date, it has been successfully applied to simple models of human cancers, namely pediatric tumors. Here the limited number of carried genetic events, due to the relative short-term latency of tumor onset, the fewer associated risk factors and the frequent development of these malignancies in non self-renewing tissues, has positively impacted on molecular characterization and, ultimately, on the diagnostic work-up of the disease [23].

The genetic characterization of adult solid tumors is more complex. The many mutational events accumulated during the disease course can indeed hamper the discrimination between driver and passenger mutations and hence the identification of the mutational landscape in these cancers.

An even more complex scenario probably occurs in CC. Although some studies have focused on the genetic characterization of CC, the overall molecular pathogenesis of this malignancy still remains poorly defined. Recent findings suggest that this process may involve a multitude of mutational events, including mutations in KRAS, TP53, BRAF, PTEN, SMAD4, IDH1, IDH2, BAP1, ARID1A and PBRMI genes, as well as FGFR translocations [24-37]. Some of these mutational events have been also implicated in CC prognosis. In particular, BAP1 and PBMR1 mutations have been associated with bone metastases and worse survival in extrahepatic CC (ECC), while KRAS and TP53 mutations, as well as loss of PTEN expression, with worse survival in intrahepatic CC (ICC) [32, 38, 39]. The prevalence of these molecular alterations, however, varies among the studies and definitive results are still lacking. Nonetheless, it is clearly emerging that ICC and ECC represent two distinct tumors arising from different genetic backgrounds [31, 32].

Accumulating evidence strongly supports the notion that the remarkable genetic heterogeneity of CC may be the result of a complex interplay among different factors, some shared by most human cancers, while others typical of this malignancy.

Recent findings suggest that the large number (probably the highest among all human malignancies) of established or putative risk factors associated with CC (Table 3) [40-47, reviewed in 48] may play a pivotal role in driving genetic heterogeneity in this disease. In this regard, a differential mutation pattern between ICC developing on chronic advanced disease and ICC arising on normal liver has been reported [34]. In particular, an opposite mutation rate (higher for EGFR and lower for KRAS, MLH1 and GNAS genes) has been observed in ICC with chronic advanced disease compared to ICC with normal liver, while PIK3CA, PTEN, CDKN2A and TP53 mutations were found only in ICC developing on normal liver. Furthermore, whole-exome sequencing analysis between CCs linked to *O. viverrini* infection and CCs not related to such exposure has identified two distinct mutational patterns in the two subgroups, with a major frequency of BAP1, IDH1 and IDH2 mutation in non-*O. viverrini*-related CCs and a major mutation rate in TP53, KRAS, SMAD4, MLL3, ROBO2, RNF43 and PEG3 genes in *O. viverrini*-related CCs, respectively [26]. Mutations of RNF43 and PEG3 genes, in particular, have been shown to activate the WNT/β-catenin signaling, leading to chromosomal instability and cell proliferation [49, 50]. Activation of this pathway has been reported in CC (particularly in ICC), a phenomenon that may be linked not only to mutations occurring in these two genes, but also to the presence of inflammatory macrophages in the stroma surrounding the tumor, which sustain WNT/β-catenin signaling through the production of WNT ligands [51, 52]. The frequent pathological activation of this pathway in CC reinforces the notion that WNT/β-catenin signaling could play a pivotal role in CC carcinogenesis and represent a potential therapeutic target in this disease, as recently reported [52].

**Table 3:** Established and putative risk factors for intra-and extrahepatic CC

Intrahepatic CC			Extrahepatic CC		
	Characteristics of the association			Characteristics of the association	
Risk factor	Strength	Level of evidence	Risk factor	Strength	Level of evidence
Bile duct cysts ^(40)^	+++	+++	Bile duct cysts ^(40)^	+++	+++^a^
PSC ^(40)^	+++^a^	+++^a^	PSC ^(40)^	+++^a^	+++^a^
Caroli's disease ^(41)^	+++^a^	+++^a^	Caroli's disease ^(41)^	+++^a^	+++^a^
Hepatolithiasis ^(40)^	+++	+++	Choledocholitihasis ^(40)^	+++	++
Choledocholitihasis ^(40)^	+++	++	Cholangitis ^(40)^	+++	++
Cholangitis ^(40)^	+++	++	O. viverrini ^(40)^	+++^a^	+++^a^
O. viverrini ^(40)^	+++^a^	+++^a^	C. sinensis ^(40)^	+++^a^	+++^a^
C. sinensis ^(40)^	+++^a^	+++^a^	Cirrhosis ^(43)^	++	++
Cirrhosis ^(43)^	+++	+++	HBV ^(44)^	+	+
HBV ^(44)^	++	+++	HCV ^(42)^	+	+
HCV ^(43)^	++	+++	Cigarette smoking ^(43)^	+	+
NASH ^(58)^	+	+	Diabetes mellitus ^(43)^	+	++
Hemochromatosis ^(45)^	?	++	Thorotrast ^(40)^	+++^a^	+++^a^
Wilson's disease ^(46)^	?	++		+	+
Cigarette smoking ^(43)^	+	+			
Alcohol ^(43)^	++	++			
Obesity ^(43)^	+	++			
Diabetes mellitus ^(43)^	+	++			
Thorotrast ^(40)^	+++^a^	+++^a^			
Asbestos ^(47)^	++	+			

Abbreviations: HBV, hepatitis B virus; HCV hepatitis C virus; NASH, non-alcoholic steatohepatitis; PSC, primary sclerosing cholangitis.

a Available studies did not distinguish between ICC and ECC

NB: + = weak association/weak evidence; ++ = moderate association/moderate evidence; +++ = strong association/strong evidence.

The induction of distinct genetic profiles in CC by different risk factors could rely not only on the different carcinogenetic mechanisms driven by such risk factors, but also on the existence along the biliary tree of two distinct stem cell niches, the canals of Hering harboring hepatic stem cells (HpSCs) and the peribiliary glands harboring biliary tree stem/progenitor cells (BTSCs) [53-55, reviewed in 56]. Cell populations from HpSCs and BTSCs lineages have been hypothesized to represent distinct candidate cells of origin (the normal cells that acquire the first cancer-initiating mutations) during CC carcinogenesis, susceptible to distinct risk factors and responsible for the development of the different ICC and ECC subtypes [53-55, reviewed in 56]. In particular, it is been suggested that the BTSC lineage may be activated under pathological conditions affecting the large intrahepatic and extrahepatic bile ducts (including liver flukes, cholangitis, primary sclerosing cholangitis, hepatolithiasis, etc.), giving rise to large bile duct pure mucin secreting ICC and ECC. Conversely, the hHpSC lineage has been suggested to be activated in response to parenchymal liver diseases (such as chronic viral/non viral liver disease, schistosomiasis and liver cirrhosis) and to be involved in the development of combined hepatocellular carcinoma-ICC, bile ductular ICC and mixed ICC (this last form being characterized by areas of focal hepatocytic differentiation, ductular reaction and mucin-secreting adenocarcinoma) [53-55, reviewed in 56, 57]. This stem cell compartment is probably activated also during non-alcoholic steatohepatitis (NASH) and asbestos exposure, as these two risk factors are exclusively associated with the development of ICC [47, 58].

The genetic characterization of CC may be further complicated by the presence of tumor clonal heterogeneity (especially in the case of mixed ICC, due to the presence of mixed differentiation features), a hallmark of almost all human cancers [59-65]. Indeed, founder cells harboring most of the tumor-borne genetic mutations typically coexist with genetically distinct tumor sub-clones, intermixed or spatially separated across the different topographic regions of the primary tumor (intratumoral heterogeneity), and the same metastasis (intrametastatic heterogeneity). Genetic variability can also occur among metastases derived from the same primary tumor (intermetastatic heterogeneity), since the genetically different sub-clones of the primary tumor can give rise to distinct lesions at the metastatic site [59-65]. This complex picture can be further exacerbated by the highly dynamic nature of the tumor itself. Tumor clonal architecture and genetic profile evolve dynamically over time (probably following a branched, Darwinian evolutionary process) under the selective pressure of the tumor microenvironment and therapeutic context, and as a consequence of genomic instability and stochastic mutational events occurring during DNA replication [59-67]. This last aspect could be particularly relevant in CC since the diagnosis, frequently late, often occurs when large macroscopic lesions have developed and tumor cells have therefore undergone many cell divisions.

The dynamic changes in time and in space of tumor subclonal composition may lead to an underestimated or biased identification of the mutational landscape in heterogeneous tumors if genetic characterization is carried out only on a single tumor biopsy [68]. This approach currently represents the standard of tumor diagnosis and the backbone of personalized therapy decisions and may significantly account for the high failure rate of some anti-cancer therapies. Sequencing of multiple tumor sites at different time points during the disease course (diagnosis, relapse and metastasis) has therefore been suggested to resolve, at least in part, spatial and temporal tumor clonal variability, and to better discriminate between mutational events in the trunk (occurring at early stages of tumor growth and detectable at all disease sites) or branches (occurring later during the development of tumor subclones and not detectable at all disease sites) of the tumor phylogenetic tree [67-69]. Such procedure would probably represent the best approach in CC, since this malignancy is very often characterized by multi-focal lesions at the primary site even at diagnosis, but unfortunately it is not devoid of risks for the patient. More recently, genotyping of circulating tumor DNA fragments (the so-called “liquid biopsy”) is emerging as a promising and less invasive type of analysis for tracking tumor dynamics and monitoring genomic evolution in real time [70]. Such DNA fragments are indeed released in the bloodstream by tumor cells undergoing apoptosis/necrosis and harbor the same genetic alterations of the tumor itself, with the advantage that sampling of the blood is minimally invasive for the patient and can be carried out at any time during disease course. Further studies are needed, however, to establish whether circulating tumor DNA genotyping could represent an effective strategy for the management of cancer patients, including CC ones, in clinical practice.

**Figure 1:**
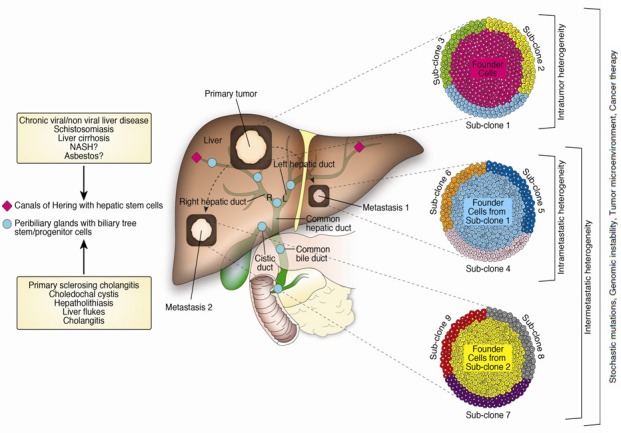
Overview of the main potential factors driving genetic heterogeneity in CC Genetic variability in CC could be the result of a complex interplay among several factors including: **a.** the existence of two genetically distinct stem cell niches along the biliary tree (the canals of Hering with hepatic stem cells and the peribiliary glands with biliary tree stem/progenitor cells), with a different susceptibly to risk factors; **b.** tumor clonal heterogeneity. Genetically distinct tumor cell sub-clones can coexist with founder cells harboring most of the tumor-borne genetic mutations, either in the primary tumor (intratumoral heterogeneity), or in the same metastasis (intrametastatic heterogeneity). Genetic variability can also occur among metastases derived from the same primary tumor, as the different sub-clones of the primary tumor can give rise to genetically distinct lesions at the metastatic site (intermetastatic heterogeneity); **c.** stochastic mutations and genomic instability. The large number of cell divisions required for cancer growth makes tumor cells prone to accumulate genomic alterations with a high frequency, due to random mutations occurring during DNA replication and deficiencies in the mechanisms involved in DNA repair; d) tumor microenvironment and cancer treatment. The tumor microenvironment and cancer therapy can induce fluctuations in tumor sub-clonal architecture and genetic profile by promoting the selective growth of sub-clones with a survival advantage within a given tumor microenvironment or a given therapeutic setting and by eradicating those with a less favorable survival advantage.

Overall, the highly complex array of causative factors (summarized in Figure 1) potentially contributing to genetic variability in CC poses a challenge in capturing the genomic landscape of this disease. Despite the great potential of NGS, this technology should be therefore applied to CC genetic characterization with caution in order to avoid possible biases and criticisms in detecting driver mutations in CC carcinogenesis. This approach is further justified by the urgent need to better define potentially actionable targets in this disease. Although it is still an unresolved issue, the mutations in molecular pathways (including KRAS, mTOR, tyrosine kinase receptor signaling) driving the carcinogenesis of many human cancers suggest that these alterations may also drive CC carcinogenesis, opening the possibility to stratify CC patients into distinct molecular subgroups for targeted therapies [31].

Given the marked genetic variability of CC, it seems likely that the best chance of response could be achieved by a combination of drugs targeting different driver mutations in this disease. Obviously, the ideal therapeutic regimen would include drugs specifically tailored to the unique genetic make-up of the individual patient, which is the cornerstone of “true” personalized therapy in cancer treatment [71]. This approach represents the final goal of future precision medicine but, unfortunately, it is still not feasible in clinical practice (even in tumors whose genetic pathogenesis is well known), mainly because of the current lack of knowledge about the toxicity of some drug interactions and the difficulty of prescribing high cost drugs whose refund cannot be guaranteed by regulatory entities. Therefore, the current selection of cancer patients likely to benefit from a specific targeted therapy is still based on tumor histology and on the analysis of a limited number of hotspot mutations.

More recently a new strategy, the so-called basket trial, suggests the use of targeted therapies exclusively on the basis of identified actionable targets, irrespective of tumor histology and other clinical/biological variables [72]. Despite the growing enthusiasm for this approach, the results of the CUSTOM basket trial have pointed out its potential limits [73]. Indeed, while a clinically significant overall response rate of 60% was reported in the subgroup of patients with the EGFR mutation who received erlotinib, the other 40% of patients did not respond, suggesting additional “hidden” oncogenic events may be responsible for treatment failure. Furthermore, selumetinib monotherapy did not achieve its primary endpoint in patients with RAS or RAF mutations. These findings strongly suggest that the disease-specific context cannot be easily bypassed, as it very often determines whether a candidate mutation represents a clinically valid endpoint. This is well known, for example, for the BRAF^V600E^ mutation, which is associated with a clinical response to BRAF inhibitors in melanoma but not in colon cancer [74, 75]. *An excellent* clinical response to *doublet-targeted therapy with the BRAF and MEK inhibitors dabrafenib and trametinib* has also been reported in an ICC patient harboring this mutation [76]. *However, whether* BRAF^V600E^ represents a driver mutation in ICC and whether other ICC patients with this mutation could benefit from treatment with BRAF inhibitors await clarification.

In summary, the identification of candidate actionable mutations in CC is challenging because of the many factors potentially contributing to genetic heterogeneity in this malignancy. Undoubtedly, NGS may offer an unique opportunity to broaden our understanding of CC molecular pathogenesis; however, a multidisciplinary approach integrating genomic, functional and clinical studies is mandatory to translate the results obtained into effective targeted therapies for this orphan disease.
